# Chinese Named Entity Recognition for Automobile Fault Texts Based on External Context Retrieving and Adversarial Training

**DOI:** 10.3390/e27020133

**Published:** 2025-01-27

**Authors:** Shuhai Wang, Linfu Sun

**Affiliations:** 1 School of Computing and Artificial Intelligence, Southwest Jiaotong University, Chengdu 611756, China; yw1688@my.swjtu.edu.cn; 2 Manufacturing Industry Chain Collaboration and Information Support Technology Key Laboratory of Sichuan Province, Southwest Jiaotong University, Chengdu 610031, China

**Keywords:** automobile fault text, named entity recognition, adversarial training, bidirectional long short-term memory, information entropy

## Abstract

Identifying key concepts in automobile fault texts is crucial for understanding fault causes and enabling diagnosis. However, effective mining tools are lacking, leaving much latent information unexplored. To solve the problem, this paper proposes Chinese named entity recognition for automobile fault texts based on external context retrieval and adversarial training. First, we retrieve external contexts by using a search engine. Then, the input sentence and its external contexts are respectively fed into Lexicon Enhanced BERT to improve the text embedding representation. Furthermore, the input sentence and its external contexts embedding representation are fused through the attention mechanism. Then, adversarial samples are generated by adding perturbations to the fusion vector representation. Finally, the fusion vector representation and adversarial samples are input into the BiLSTM-CRF layer as training data for entity labeling. Our model is evaluated on the automotive fault datasets, Weibo and Resume datasets, and achieves state-of-the-art results.

## 1. Introduction

With the rapid development of the world economy and the increasing number of cars, car repair services have an increasingly important role in after-sales services. Under such circumstance, it is important to know about past faults. Currently, most of the information about car failures is recorded in the form of texts, which contain a large amount of automotive repair knowledge. To effectively utilize these textual data, we need to transform them into structured and applicable knowledge. Named entity recognition (NER) plays a crucial role in this transformation by identifying key entities of the text, such as fault locations, the root causes of faults, and repair techniques.

Some existing studies have achieved good results in Chinese NER, but they cannot be applied to identify the various entities required in automotive fault texts. The study of named entity recognition in automobile fault texts is different from the study of named entity recognition in general domain texts [1]. Named entities in generic texts are written in a standardized manner and named in a strict manner [2]. However, the automobile fault texts are elliptical and colloquial, which means the fault events recorded by the maintainers do not necessarily follow the standard grammatical structure (e.g., subject–verb–object structure) and some records may have no subjects or predicates [3]. The entities in automobile fault texts contain many specialized terms. For instance, ‘ECU’ refers to the electronic control unit, ‘ABS’ denotes the anti-lock braking system, and ‘DPF’ represents the diesel particulate filter. These terms frequently appear in the text, but without understanding their meaning, it is challenging to identify them correctly. Take another example, the sentence “后桥支架 (Rear Axle Support)”: “The rear axle bracket” is a component of the car, which refers to the bracket installed on the rear axle, rather than referring to the two entities “rear axle” and “bracket”. It is challenging to determine whether “后桥支架 (Rear Axle Support)” is an entity and where the boundary of the entity is during the entity extraction process.

Therefore, the elliptical and colloquial language characteristics, along with the presence of numerous domain-specific terms, make it challenging to effectively identify key concepts in automotive fault texts. Most previous methods have achieved good results. However, it is difficult to accurately identify key concepts in automotive failure texts, especially for sparse and emerging terms, as previous studies have not been able to effectively capture contextual information.

To solve above issues, this paper proposes a Chinese named entity recognition based on external contextual retrieval and adversarial training (CNEREA). The model generates externally relevant texts semantically related to the original input sentence by utilizing an improved keyword extraction method, BM25, and a search engine. Then, the original sentence and its external texts are fed into the Lexicon Enhanced BERT (LEBERT) separately to obtain the character–word fusion embedding representation, respectively. The character–word fusion embedding representation refers to the integration of word information into a character-based embedding representation. Next, the character–word fusion embedding representations are fused using the attention mechanism. Finally, the long-distance semantic information is extracted using a bidirectional long short-term memory network (BiLSTM), and the recognition results are obtained using Conditional Random Field (CRF). During the training process, we introduce an adversarial training to enhance the robustness and generalization of the proposed model by adding adversarial samples.

The rest of the paper is organized as follows. Section 2 reviews the related works in Chinese NER, pre-training model, and adversarial training. In Section 3, we describe the CNEREA. In Section 4, we verify the validity of the CNEREA. Section 5 concludes the paper.

## 2. Related Work

In this section, we discuss three categories of related works about NER, the pre-training model and adversarial training.

### 2.1. Named Entity Recognition

The NER task is to identify concepts with specific meanings from texts [3]. These concepts are also called named entities (i.e., NE), which are generally divided into two categories: generic NEs (e.g., person, location, and organization) and domain-specific NEs (e.g., terminology) [4]. NER can be classified into three categories: rule-based and template-based methods, traditional machine learning methods, and deep learning methods [5,6]. Specifically, rule-based and template-based methods rely on manually constructed rules and templates. These methods not only consume a significant amount of manpower and resources but also suffer from poor transferability and generalization capabilities. In contrast, traditional machine learning methods train models using annotated datasets. Common methods encompass maximum entropy models, support vector machines, hidden Markov models, and Conditional Random Fields. However, these methods are prone to issues such as error propagation. Consequently, with the advancement of technology, deep learning-based methods have gradually become a research hotspot in the field of NER.

Unlike English, the Chinese language exhibits a distinctive characteristic in which the demarcation between words lacks clarity, along with an intricate grammatical structure and numerous synonyms [1,3,7]. To avoid word segmentation errors, some approaches [8,9] perform Chinese NER directly at the character level. However, character-based methods fail to fully utilize the available lexical information and the sequential information between words [10]. Therefore, some scholars have introduced dictionary information into character-based Chinese NER models to enhance the performance of entity recognition [7,11]. Zhang et al. [12] proposed a lattice-structured LSTM (Lattice-LSTM) model for Chinese NER. This model encodes all characters and potential words recognized by a lexicon in a sentence, avoiding the error propagation of segmentation while leveraging the word information. Li et al. [13] proposed the Flat-Lattice Transformer (FLAT) model for Chinese NER. By transforming the complex character–word lattice structure into a flat structure and designing a specific position encoding, FLAT effectively improves the performance and efficiency of Chinese NER, while achieving faster inference speeds. Li et al. [14] introduced a named entity recognition method based on enhanced lexical features (NER-ELF) for cultural relics. This method introduces a multi-level temporal convolutional module at the encoding layer to encode the text and capture lexical features of different granularities.

In addition to lexical information, other external information has also been utilized to semantically enhance Chinese NER. Wang et al. [15] proposed a Chinese NER model named Multi-Embedding Global Pointer (MEGP), which improves performance by combining radical and pronunciation features. The model combines these features with a global pointer network to improve Chinese NER. Zhang et al. [16] introduced a novel Chinese NER model called VisPhone, which aims to enhance the model’s capability to capture contextual semantics by incorporating the phonetic and visual information of Chinese characters into the model. Wang et al. [17] proposed an improved NER model that enhances performance by retrieving relevant contexts from a search engine. Furthermore, to increase the model’s robustness in the absence of external contexts, the model employs a Cooperative Learning method based on the L2 norm, termed CL-L2.

The above studies have achieved good results. Automotive failure texts are elliptical and colloquial, and contain a large number of domain-specific terms, making it difficult for traditional methods to effectively identify key concepts in them. Although NER models based on lexical information and other external information mentioned above are able to identify some key concepts, these models perform poorly in accurate recognition due to the lack of sufficient contextual information. Especially for rare and emerging terms, the lack of available context makes it more difficult for the above methods to effectively identify key concepts of automotive faults. This paper utilizes a search engine to retrieve and select some semantically related external contexts for improving the recognition of key concepts in automotive fault texts.

### 2.2. Pre-Training Models in Chinese NER

Transformer-based pre-trained models, such as BERT [18], have shown excellent performance for Chinese NER. Some scholars have combined the advantages of lexical information and pre-trained models for exploring Chinese NER. Hu et al. [19] proposed a method that combines the pre-trained BERT model with the CRF, thereby constructing the BERT-CRF model, with the aim of enhancing the performance of entity recognition. Mengge et al. [20] proposed the Porous Lattice Transformer Encoder for Chinese NER. It enhances self-attention to incorporate the lattice structure and introduces a porous mechanism to augment localness modeling while retaining the strength of capturing rich, long-term dependencies. However, existing methods [12,13,21,22] only fuse lexical features through a shallow random initialization sequence layer without integrating them into the underlying layer of BERT. In order solve the problem, Liu et al. [23] proposed the LEBERT model, which integrates external lexicon knowledge directly into the BERT layer via a lexicon adaptation layer.

In order to fully utilize lexical information, this paper adopts LEBERT to enhance the model’s ability to capture the semantic and boundary features of words, thereby significantly improving the effectiveness of entity recognition.

### 2.3. Adversarial Training for NER

In recent studies, many researchers have applied adversarial training to the field of natural language processing and achieved good results. Adversarial training, as a form of regularization, improves the robustness of the model by inputting disturbances into the model. For Chinese NER tasks in the rail field, Su et al. [3] proposed a novel adversarial training-based lattice LSTM model. The model applies lattice LSTM and CRF to achieve Chinese NER, and further improves the performance of the model in recognizing train fault information through adversarial training. Liang et al. [24] proposed a novel model for the named entity recognition of Chinese crop diseases and pests, which integrates RoBERTa-wwm-BiGRU-CRF and adversarial training. In the training process, adversarial perturbation is added at the word vector layer to improve the model’s generalization ability and the recognition performance of entities with unclear boundaries. Ma et al. [25] introduced a method for named entity recognition in scientific and technical texts that is based on pre-trained models and adversarial training. This method employs the Fast Gradient Method (FGM) for semi-supervised NER, effectively enhancing the identification of named entities in the scientific literature. Cheng et al. [2] proposed a BERT-based medical dialogue named entity recognition method, BERT-BiLSTM-CRF-ADV, which utilizes the BERT pre-training model to obtain rich semantic word vectors, employs BiLSTM for feature extraction, and applies CRF for constraint correction.

This paper applies adversarial training to the training of the automotive fault NER model. Perturbation factors are added to the the character–word embedding representation of the model to generate adversarial samples. The generated adversarial samples can enhance the robustness of the model.

## 3. Methodology

**Figure 1:**
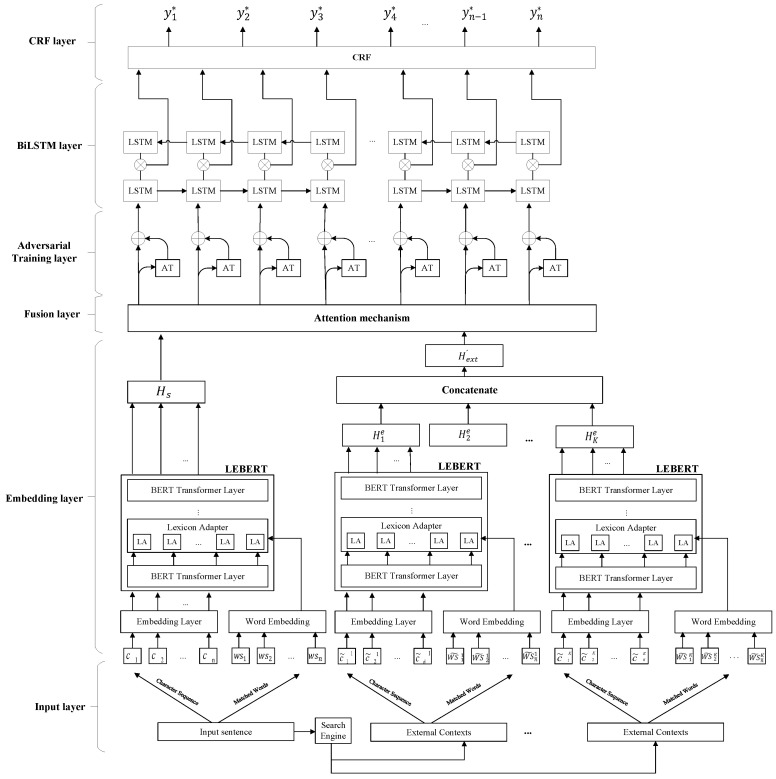
The overall schema of the proposed model.

In this section, we introduce CNEREA. CNEREA consists of an input layer, embedding layer, fusion layer, adversarial training layer, BiLSTM layer, and CRF layer, as shown in Figure 1.

### 3.1. Input Layer

Our approach retrieves external knowledge by an off-the-shelf search engine [26]. We extract *m* query keywords from the original input sentence using a keyword extraction method. The extracted keywords subsequently serve as query criteria to obtain relevant textual data from the search engine [17]. We then adopt the BM25 algorithm to re-rank the retrieved texts according to their semantic similarity to the original input sentence and choose the top-*K* texts as external related texts $\tilde{s}=\left\{{\tilde{s}}_{1},{\tilde{s}}_{2},\ldots ,{\tilde{s}}_{\tilde{i}},\ldots ,{\tilde{s}}_{k}\right\}$, where *K* is the number of external related texts.

TextRank [27] is a graph-based algorithm which uses co-occurrence relationships between words to calculate the importance of words and selects keywords based on the importance ranking. BM25 [28] is a widely used text similarity algorithm based on term frequency as appeared in the corpus. These two statistic-based unsupervised approaches utilize word frequency to calculate semantic relevance, rather than semantic generation using word embedding, in order to prevent the information leakage of named entities [26]. Since BM25 is widely used, we only introduce the improved TextRank.

The TextRank algorithm is a graph-based ranking model primarily used for keyword extraction and text summarization in natural language processing. It draws inspiration from Google’s PageRank algorithm, assessing the importance of words or sentences in a text based on their relationships with one another. We consider each automobile fault repair record as a collection of words, where each word in the repair record is treated as a node in a graph. The importance of a node is determined by the number of neighboring nodes that point to it. Therefore, the weight $S\left(ws_{i}\right)$ of a node ws is calculated as follows:

(1) $$S\left(ws_{i}\right)=\left(1-d\right)+d\times \sum_{ws_{j}\in \mathrm{In}\left(ws_{i}\right)}\frac{\omega_{ij}}{\sum_{ws_{k}\in \mathrm{Out}\left(ws_{j}\right)}\omega_{jk}}S\left(ws_{j}\right)$$

where $\mathrm{In}\left(ws_{i}\right)$ represents the set of nodes pointing to $ws_{i}$; $\mathrm{Out}\left(ws_{j}\right)$ is the set of nodes pointed by $ws_{j}$; *d* is a damping coefcient, and is set to 0.85 by default.

As the texts of automotive fault records are generally short, keywords tend to appear infrequently within individual records. But they may occur more frequently in the entire dataset. If the dataset-based word frequency values are directly used to improve the TextRank algorithm, the scores of low-frequency words will be significantly lower than those of high-frequency words. To solve this problem, this paper introduces weighted information entropy to optimize the TextRank algorithm. Information entropy is used to represent the value of information. A higher entropy value indicates that a word contains more information. The weighted information entropy is defined as follows:

(2) $$H\left(ws_{i}\right)=\lambda \times \sum_{k=1}^{\overset{`}{N}}\frac{{\tilde{N}}_{k}^{i}}{{\overset{˘}{N}}^{i}}\log_{2}\left(\frac{{\overset{˘}{N}}^{i}+1}{{\tilde{N}}_{k}^{i}}\right)$$

where ${\tilde{N}}_{k}^{i}$ denotes the word frequency of word $ws_{i}$ in the *k*th car repair record, ${\overset{˘}{N}}^{i}$ denotes the word frequency of word $ws_{i}$ in the dataset, and $\overset{`}{N}$ denotes the total number of car repair records in the dataset. λ is the weight obtained by the part-of-speech of word $ws_{i}$.

According to TextRank, the transfer probability between nodes is shown as follows:

(3) $$W_{\mathrm{Entropy}\;}\left(ws_{j},ws_{i}\right)=\frac{H\left(ws_{i}\right)}{\sum_{ws_{k}\in \mathrm{Out}\left(ws_{j}\right)}H\left(ws_{k}\right)}$$

According to Equation (3), the modified $S\left(ws_{i}\right)$ is shown as follows:

(4) $$S\left(ws_{i}\right)=\left(1-d\right)+d\times \sum_{ws_{j}\in \mathrm{In}\left(ws_{i}\right)}W_{\mathrm{Entropy}\;}\left(ws_{j},ws_{i}\right)S\left(ws_{j}\right)$$

Referring to the keyword extraction method described in reference [29], an improved keyword extraction method (IKEM) is designed that combines the advantages of TF-IDF and TextRank to improve the accuracy and efficiency of keyword extraction. For a more detailed explanation of the TF-IDF method, please refer to reference [29]. The following is a description of the improved keyword extraction method.

Input: Text *T*, number of keywords *m*, maximum number of iterations *D*, threshold ϵ, damping factor d=0.85, average number of keywords in the manually annotated dataset is $\bar{n}$.

Output: Keyword *S*.

Step 1: Perform word segmentation, remove stop words, and conduct part-of-speech tagging on the text *T* to obtain the word list.

Step 2: Filter the word list to retain nouns, verbs, and gerunds, forming an initial set of candidate keywords list KeList.

Step 3: Calculate $H(ws_{i})$ for each word in KeList according to Equation (2). $H(ws_{i})$ is used as the initial weight of each word node.

Step 4: Utilize Equation (4) for multiple iterations to compute the weight $S\left(ws_{i}\right)$ of each candidate keyword until convergence. The convergence condition is that the weight change of all nodes is less than ϵ, or the maximum number of iterations *D* is reached.

Step 5: Sort the weights *S* of the candidate keywords and select the top *m* keywords as the extraction results $KWords_{C}$.

Step 6: Use TF-IDF to extract keywords from KeList to obtain keyword set $KWords_{A}$.

Step 7: Use the traditional TextRank from KeList to obtain keyword set $KWords_{B}$.

Step 8: $KWords_{H}=KWords_{A}\cup KWords_{B},KWords_{W}=KWords_{H}\cap \;KWords_{C}=\left\{ws_{1},ws_{2},\ldots ,ws_{\tilde{n}}\right\}$.

Step 9: If $\tilde{n}\geq \bar{n}$, then $S=KWords_{W}$; conversely, if $\tilde{n}\geq 0$ and $\tilde{n}<\bar{n}$, then $S=KWords_{C}$.

### 3.2. Embedding Layer

Traditional NER methods use character-level tagging (BIOES), but character sequences convey limited semantic information [30]. To enhance the expressiveness of the embedding layer, we integrate lexical information through LEBERT, which applies the Lexical Adapter (LA) to a specific BERT layer. For LA details, see reference [23]. Below, we introduce LEBERT.

Given a Chinese dictionary D and a Chinese sentence $s=\left\{c_{1},c_{2},\ldots ,c_{i},\ldots ,c_{n}\right\}$ comprising of *n* characters, we construct the corresponding sequence of character–word pairs $rs=\left\{\left(c_{1},ws_{1}\right),\left(c_{2},ws_{2}\right),\ldots ,\left(c_{n},ws_{n}\right)\right\}$ according to the method described in the reference [23]. The input *s* is first passed into the input embedder, which outputs $E=\left\{e_{1},e_{2},\ldots ,e_{n}\right\}$ by adding token, segment, and position embeddings. Then, we input *E* into the Transformer encoder, and each Transformer layer is as follows:

(5) $$GL=LN\left(H^{l-1}+\mathrm{MATT}\left(H^{l-1}\right)\right)$$

(6) $$H^{l}=LN\left(GL+\mathrm{TFFN}\left(GL\right)\right)$$

where $H^{l}=\left\{h_{1}^{l},h_{2}^{l},\ldots ,h_{n}^{l}\right\}$ is the output of the *l*-th layer; $H^{0}=E$; MATT denotes the multi-head attention mechanism; TFFN denotes a two-layer feed-forward network which uses ReLU as hidden activation function; and LN is layer normalization.

Assume that lexical information is integrated between the $\tilde{k}$-th and ($\tilde{k}$ + 1)-th transformer layers. To inject lexical information, the output $H^{\tilde{k}}=\left\{h_{1}^{\tilde{k}},h_{2}^{\tilde{k}},\ldots ,h_{n}^{\tilde{k}}\right\}$ is obtained after passing through $\tilde{k}$ successive Transformer layers. Subsequently, the Lexical Adapter processes each pair $\left(h_{i}^{\tilde{k}},x_{i}^{ws}\right)$ and converts the *i*-th pair into ${\tilde{h}}_{i}^{\tilde{k}}$:

(7) $${\tilde{h}}_{i}^{\tilde{k}}=\mathrm{LA}\left(h_{i}^{\tilde{k}},x_{i}^{ws}\right)$$

In BERT, the number of transformer layers *M* is set to 12. We input ${\tilde{H}}^{\tilde{k}}$ into the remaining ($M-\tilde{k}$) Transformers. Finally, we obtain the output of the *M*-th Transformer, denoted as $H^{m}=\left\{{\tilde{h}}_{1},{\tilde{h}}_{2},\ldots ,{\tilde{h}}_{i},\ldots ,{\tilde{h}}_{n}\right\}$. In the same way, input *K* external related texts $\tilde{s}$ to multiple LEBERTs and output $H_{\mathrm{ext}}=\left\{H_{1}^{e},H_{2}^{e},\ldots ,H_{\overset{ˇ}{i}}^{e},\ldots ,H_{k}^{e}\right\}$. In order to integrate the information of multiple external related texts, we adopt an operation that concatenates these texts:

(8) $$H_{\mathrm{ext}}^{\prime }=\left[H_{1}^{e}⊕H_{2}^{e}⊕\ldots ⊕H_{k}^{e}\right]$$

where $H_{\mathrm{ext}}^{\prime }$ and ⊕ mean the final external representation and concatenation operations, respectively.

### 3.3. Fusion Layer

In the layer, we use the attention mechanism to fuse $H_{s}$ and $H_{\mathrm{ext}}^{\prime }$, which generate fused semantic representation. For $H_{s}$, we compute attention scores over $H_{\mathrm{ext}}^{\prime }$ to generate context embedding $H_{\mathrm{context}}$ [26]. This process integrates external knowledge that is semantically relevant to the original input, thereby enhancing the context representation. It is shown below:

(9) $$H_{\mathrm{context}}=\mathrm{Attention}\left(H_{s},H_{\mathrm{ext}\;}^{\prime }\right)$$

The fused semantic representation $H_{\mathrm{fusion}}$ is obtained by computing the weighted sum of $H_{s}$ and $H_{\mathrm{fusion}}$:

(10) $$H_{\mathrm{fusion}}=q\times H_{s}+\left(1-q\right)\times H_{\mathrm{context}}$$

where the fusion factor *q* represents the weight of input embedding, and the fusion factor (1−q) represents the weight of context embedding.

### 3.4. BiLSTM Layer

BiLSTM is effective in capturing contextual information as demonstrated in previous studies [2,31]. Consequently, this paper employs BiLSTM to fully utilize the contextual information from $H_{fusion}$ and the adversarial sample $H_{adv}$ as presented in Equations (11) and (12). For a detailed adversarial sample, see the “Adversarial Training” Section 3.6.

(11) $$H\in \left\{H_{fusion},H_{adv}\right\}$$

(12) $$B_{\mathrm{bm}}=\left[\vec{\mathrm{LSTM}}\left(H\right);\overset{\leftarrow }{\mathrm{LSTM}}\left(H\right)\right]$$

where $B_{\mathrm{bm}\;}=\left\{b_{1},b_{2},\cdots ,b_{i},\cdots ,b_{n}\right\}$ denotes the output of BiLSTM.

### 3.5. CRF Layer

The CRF layer is used at the top of the BiLSTM layer [32,33]. The output sequence $B_{\mathrm{bm}\;}=\left\{b_{1},b_{2},\cdots ,b_{i},\cdots ,b_{n}\right\}$ generated by the BiLSTM is used as input for the CRF. It corresponds to the tag sequence $Y=\left\{y_{1},y_{2},\cdots ,y_{n}\right\}$. The score of the tag sequence *Y* can be computed in the following manner:

(13) $$\mathrm{score}\left(B_{\mathrm{bm}},Y\right)=\sum_{i=0}^{n}A_{y_{i},y_{i+1}}+\sum_{i=1}^{n}P_{i,y_{i}}$$

where $A\in R^{\left(n+2)\times (n+2\right)}$ represents the transfer matrix; $A_{i,j}$ signifies the transfer score from the *i*-th tag to the *j*-th tag; $P_{i,j}$ indicates the score obtained from the *j*-th tag for the *i*-th character. The probability of the label sequence *Y* is obtained by applying the softmax activation function as shown in Equation (14):

(14) $$P\left(Y|B_{\mathrm{bm}\;}\right)=\frac{\exp\left(\mathrm{score}\left(B_{\mathrm{bm}\;},Y\right)\right)}{\sum_{\tilde{y}\in Y_{B_{\mathrm{bm}\;}}}\exp\left(\mathrm{score}\left(B_{\mathrm{bm}\;},\tilde{y}\right)\right)}$$

where $Y_{B_{\mathrm{bm}}}$ represents all possible tag sequences, and $\tilde{y}$ indicates the true tag value.

The final predicted sequence result $y^{*}$ obtained using the Viterbi algorithm is the sequence with the highest total prediction score among all sequences as illustrated in Equation (15):

(15) $$y^{*}=\underset{\tilde{y}\in Y_{B_{\mathrm{bm}\;}}}{\arg\max}\mathrm{score}\left(B_{\mathrm{bm}\;},\tilde{y}\right)$$

### 3.6. Adversarial Training

Adversarial training is a technique that incorporates noise into the training process to regularize the model parameters and enhance the robustness of deep learning models [34]. In this section, we augment the training process using an adversarial training method from reference [35]. We generate the adversarial sample $H_{adv}$ by adding perturbation $\eta_{at}$ to $H_{\mathrm{fusion}}$. It is calculated as follows:

(16) $$H_{adv}=H_{\mathrm{fusion}}+\eta_{at}$$

(17) $$\eta_{at}=\frac{\varepsilon g}{∥g∥}$$

(18) $$g=\nabla_{H}\mathrm{loss}\left(H_{\mathrm{fusion}\;},Y;\theta \right)$$

where ε is a small bounded norm considered a hyperparameter; *g* is the gradient of the loss function; θ represents the learned parameters; loss(·) is the cross-entropy loss.

Then, the adversarial sample $H_{adv}$ is fed into the model (BiLSTM+CRF) to obtain the adversarial cross-entropy loss $L_{adv}$, and $H_{\mathrm{fusion}}$ is fed into the model to obtain the original cross-entropy loss $L_{pri}$. The final optimized loss *L* is as follows:

(19) $$L=L_{pri}+L_{adv}$$

(20) $$L_{pri}=\mathrm{loss}\left(H_{\mathrm{fusion}},Y;\theta \right)$$

(21) $$L_{adv}=\mathrm{loss}\left(H_{adv},Y;\theta \right)$$

## 4. Experiments and Results

### 4.1. Datasets

To verify the performance of the proposed model, we obtain car repair records from the ASP/SaaS-based manufacturing industry value chain collaboration platform [36]. These records contain four specific types of entities: fault location (FL), Fault Phenomenon (FP), fault reason (FR), and repair strategy (RS). We extract the car repair records of factory L to form dataset D1; at the same time, we extract the car repair records of factory C to form dataset D2. We extract the car repair records of factory T to form dataset D3, which includes uncommon or new fault codes, emerging part names, or descriptions of specific fault phenomena. For the D3 dataset, the division of the dataset is conducted based on temporal attributes. Precisely, data with earlier timestamps are designated as the training set, whereas data with more recent timestamps are allocated to the testing and validation sets. We use stratified sampling to preserve the category ratio of samples as much as possible [3]. And we evaluate CNEREA on D1, D2 and D3. In order to verify the generalization ability of the model, we also use two Chinese NER datasets, including Weibo NER [11,37], and Resume NER [12]. Weibo NER and Resume NER are from social media and resumes, respectively. Table 1 shows the statistic information of these datasets.

### 4.2. External Retrieval

Query generation is carried out by using the improved keyword extraction method. We adopt the Bing search API as the off-the-shelf search engine to retrieve external relevant texts. BM25 from rank_bm25 [38] is used for text similarity re-ranking. We select the top three relevant texts as the external context of the input sentence. If the total sub-token lengths of the input sentence and external contexts exceeds 300, the external context is chunked.

### 4.3. Experimental Parameters and Evaluation Metrics

The main hyperparameter settings for the CNEREW model are as follows: the batch size is set to 16, the LSTM hidden size to 128, the dropout rate to 0.5, the learning rate to $5\times 10^{-5}$, and the number of epochs to 30. And we keep some parameters the same with LEBERT [23], including the number of transformer layers, word embedding size, and so on. The layer number $\tilde{k}$ at which the Lexicon Adapter is connected is set to 1, during model training, and the Adam optimizer is adopted. Following the prior work [39,40], we choose precision (P), recall (R), and F1 as the evaluation indicators.

### 4.4. Comparison of Keyword Extraction Methods

**Figure 2:**
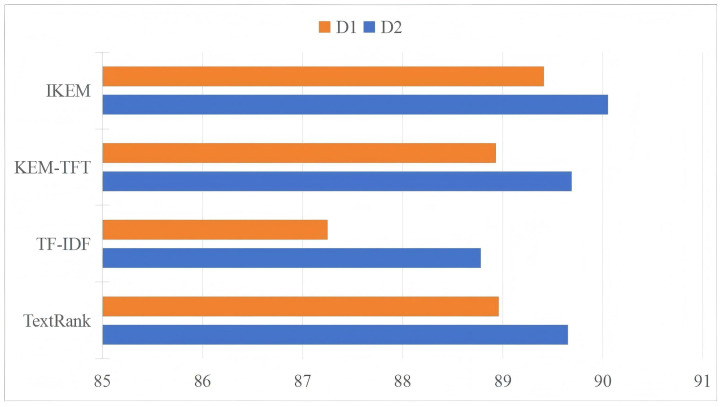
Comparison of different keyword extraction methods by F1-scores on D1 and D2.

Various keyword extraction approaches may affect the token representations of the model. We compare our approach with three other keyword extraction approaches. The first and second are the traditional TF-IDF and TextRank. These two approaches have been widely used in previous research [17,41,42]. The third is the keyword extraction method based on TF-IDF and TextRank (KEM-TFT) [43]. We extract keywords using the three keyword extraction methods, and then utilize the BM25 algorithm to obtain external contexts based on these keywords for training the proposed model. The results are presented in Figure 2. The experimental results show that the improved keyword extraction method has the best performance, indicating that it helps to improve the model performance. While TF-IDF has the lowest F1 value, this is because relying solely on word frequency to extract keywords results in some low-frequency but important keywords being ignored.

### 4.5. Ablation Study

CNEREA includes the LEBERT-BiLSTM-CRF, along with enhancements from external knowledge and adversarial training. We use the LEBERT-BiLSTM-CRF model as the basic model. The basic model with added external knowledge is denoted as “+EK”, and the basic model with added adversarial training is denoted as “+AT”. The ablation experiment results are shown in Table 2.

**Figure 3:**
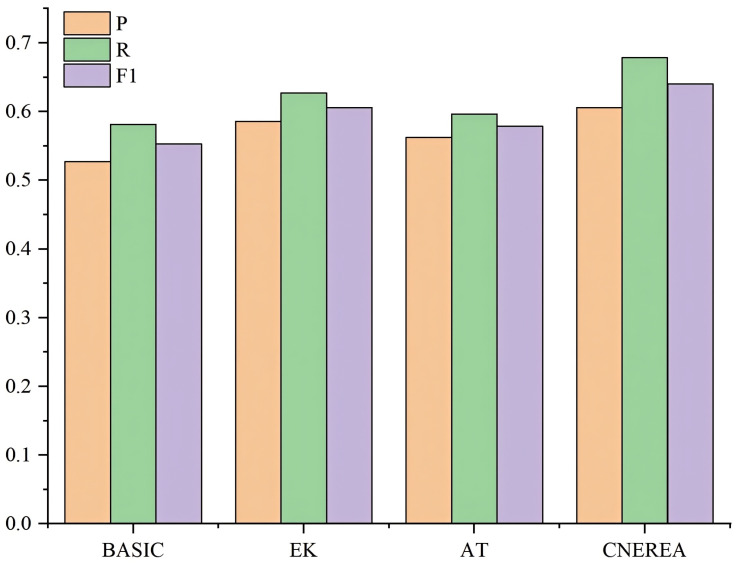
Ablation experimental results on D3.

From Table 2 and Figure 3, “+EK” outperforms the basic model in P, R, and F1. This shows that the enhancement of external knowledge is more helpful in enriching the contextual information of automobile fault texts. “+AT” adds adversarial training compared to the basic model, which leads to a significant improvement in all metrics. This improvement not only enhances the model’s ability to identify entities but also improves the model’s robustness. CNEREA is higher than “EK” in P and F1, further indicating that adversarial training contributes to improving the model’s performance and robustness. It also surpasses “+AT” across P and F1, further confirming the effectiveness of external knowledge integration.

From Figure 3, it is obvious that the P, R, and F1 values of CNEREA and “EK” are significantly better than those of the other algorithms on the D3 dataset. This is because CNEREA and “EK” utilize search engines to obtain external context, allowing them to more effectively identify rare and emerging entities in the D3 dataset. For example, “…先检查制动盘，GPF，再看看制动液。… (…Check the brake disc, GPF, and then the brake fluid. …)”. Basic and AT were not able to identify the fault location, i.e., the Gasoline Particulate Filter (GPF). CNEREA and EK used a search engine to find the relevant introduction of GPF and used it as an external context to identify the fault location effectively.

### 4.6. Performance on Different Entities

**Figure 4:**
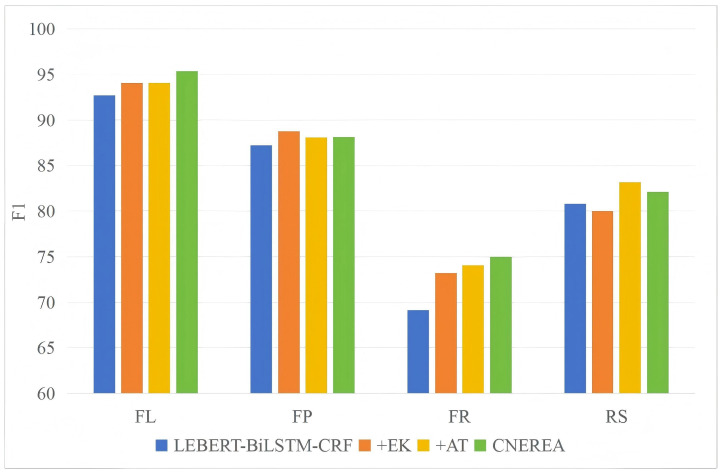
F1-scores of different entity types on D1 (%).

**Figure 5:**
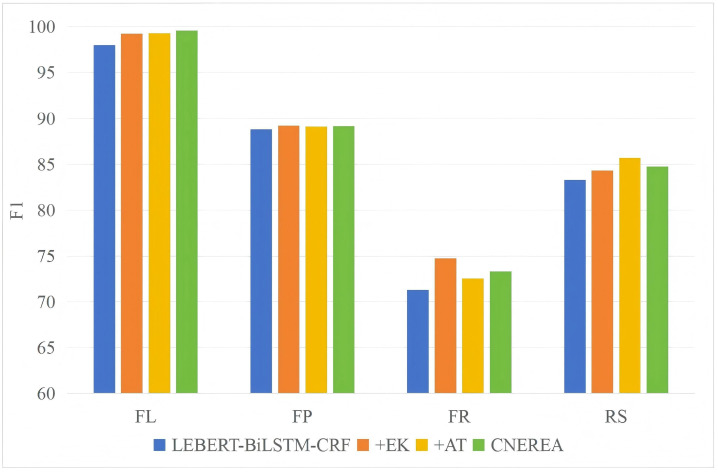
F1-scores of different entity types on D2 (%).

Figure 4 and Figure 5 shows the model performance of different entity types on D1 and D2. When the model structure changes, the specific F1 values of different entity types are also different [35]. “+EK” has shown a significant improvement effect on different entity types, especially in the performance of FR and RS entities. This is because D1 and D2 may contain some special terms and abbreviations, and these terms and abbreviations can find relevant definitions or contextual information through search engines, thereby improving the performance of entity recognition. “+AT” improves the recognition performance of almost all types of entities, indicating the effectiveness of adversarial training. In addition, the recognition performance of FR and RS is significantly lower than that of FL and FP. This phenomenon is attributed to the lack of relevant entries for fault reasons in the constructed vocabulary, which hinders the model’s ability to perform effective vocabulary enhancement. Additionally, the descriptions of fault reasons and repair strategies in the text are diverse and lack clear structural features; for instance, words with distinct structural features, such as "反应 (reflect)" in fault phenomena and "更换 (replace)" in repair strategy, not universally present. This variability in expression poses challenges for the model in identifying failure causes and repair strategy entities, thereby affecting overall performance.

### 4.7. Effect of Adversarial Training

**Figure 6:**
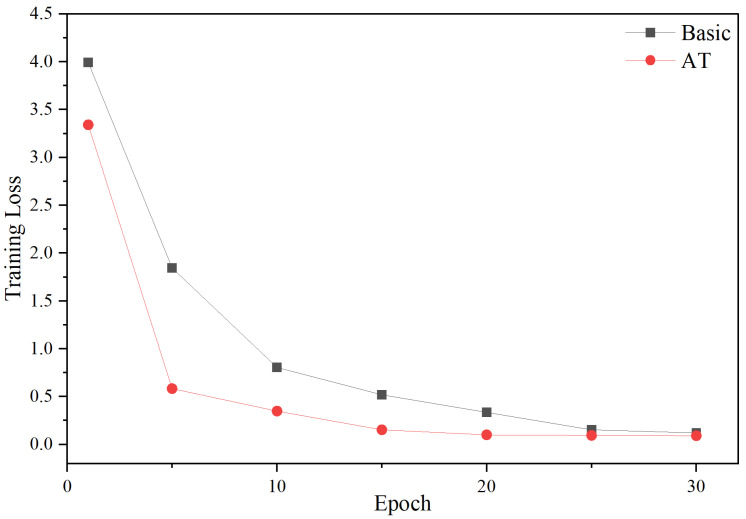
The curves of training loss on D1.

**Figure 7:**
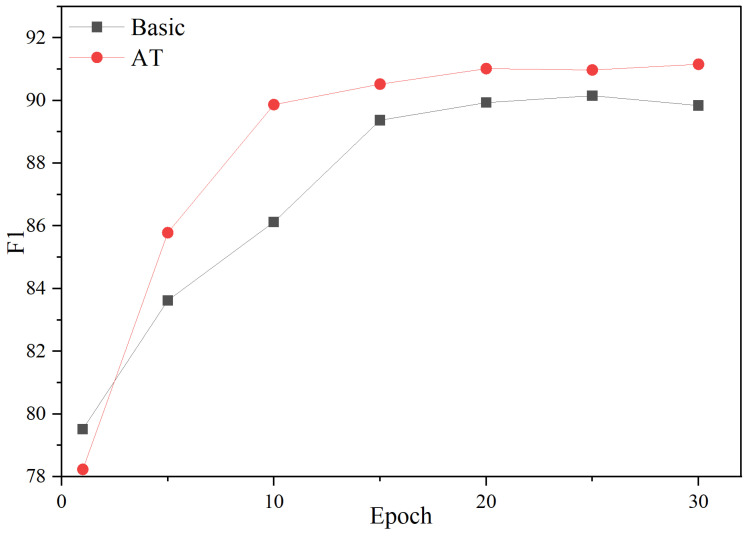
The indicators of training process on D1 with and without AT (%).

According to the analysis results of Figure 6 and Figure 7, it can be seen that adversarial training (AT) has a positive impact on model performance. As can be seen from Figure 6, the model using AT shows a faster loss reduction rate during the initial 15 epochs, reflecting the efficiency of the optimization process. Additionally, Figure 7 shows that “+AT” has a faster F1 growth rate and higher F1 value compared to the base model. This shows that adversarial training can improve the robustness and reliability of the model in practical applications.

### 4.8. Model Generalization

In order to verify the generalization ability of CNEREA, we conduct comparative experiments with other methods on the D1, D2, Resume and Weibo datasets. These comparison methods include BiLSTM-CRF [44], Lattice-LSTM [12], BERT-CRF [19], FLAT-Lattice [13], CL-L2 [17], LEBERT [23], NER-ELF [14], MEGP [15], and Visphone [16]. The results are summarized in Table 3. In Table 3, CNEREA achieves an F1-score of 89.41% on D1, outperforming the leading models, LEBERT and CL-L2, by 6.34% and 6.91%, respectively. Table 3 shows that on the D2 dataset, CNEREA reaches an F1-score of 90.05%, surpassing LEBERT and CL-L2 by 5.77% and 5.84%. Additionally, on the Resume and Weibo datasets, CNEREA records notable F1-scores of 97.03% and 71.67%, exceeding other comparison algorithms. These results demonstrate the effectiveness of CNEREA in entity recognition tasks across different domains, demonstrating its generalization ability.

## 5. Conclusions

This paper proposes a CNEREA for Chinese NER tasks in the automobile fault texts. The model utilizes external knowledge to enhance the semantics of automotive fault text, which is generated using an improved keyword extraction method, BM25, and a search engine. During the training process, we introduce an adversarial training to enhance the robustness and generalization of the proposed model by generating adversarial samples. Experiments are conducted on three Chinese datasets of automotive fault texts, and the results show that CNEREA performs better than other models and significantly improves the performance of Chinese NER. Additionally, the performance of CNER on Chinese datasets from three distinct domains significantly outperforms other comparative models, indicating that the proposed model has good applicability. Besides lexical information, the effectiveness of identifying automobile fault entities may also be influenced by features such as Chinese character shapes and pronunciation. In the future, we plan to incorporate these features to further study their influence on the model’s recognition performance. Furthermore, we will apply the proposed model to the healthcare and e-commerce domains to further validate its effectiveness and applicability.

## Figures and Tables

**Table 1:** Statistics of four benchmarking datasets.

Dataset	Type	Train	Test	Dev
D1	Sentence	3.6 k	0.45 k	0.45 k
D2	Sentence	4.4 k	0.55 k	0.55 k
D3	Sentence	3.2 k	0.4 k	0.4 k
Weibo	Sentence	1.4 k	0.27 k	0.27 k
Resume	Sentence	3.8 k	0.46 k	0.48 k

**Table 2:** Ablation experimental results (%).

	Weibo			Resume			D1			D2		
Models	P	R	F1	P	R	F1	P	R	F1	P	R	F1
Basic	70.75	70.01	70.38	96.02	96.7	96.36	85.11	88.19	86.62	86.21	88.82	87.01
+EK	71.10	71.78	71.44	96.45	96.97	96.71	86.97	89.94	88.43	88.91	89.63	89.27
+AT	71.29	**71.83**	71.56	96.51	97.05	96.78	87.08	90.78	88.89	89.09	**90.95**	90.01
CNEREA	**72.68**	70.69	**71.67**	**96.60**	**97.46**	**97.03**	**87.69**	**91.20**	**89.41**	**89.27**	90.84	**90.05**

The best results are shown in bold.

**Table 3:** Results on different datasets (%).

	Weibo			Resume			D1			D2		
Models	P	R	F1	P	R	F1	P	R	F1	P	R	F1
BiLSTM-CRF	68.8	49.3	57.4	94.53	94.29	94.41	83.14	78.93	80.98	83.36	78.93	81.08
Lattice-LSTM	53.04	62.25	58.79	94.18	94.11	94.46	85.47	80.41	82.86	84.35	82.78	83.56
FLAT-Lattice	-	-	63.42	-	-	94.93	-	-	-	-	-	-
BERT-CRF	70.33	66.11	68.13	95.49	96.07	95.78	86.03	80.27	83.05	86.66	83.72	84.68
CL-L2	69.85	68.18	69.01	**96.97**	96.2	96.59	86.53	80.91	83.63	86.04	84.15	85.08
MEGP	70.24	**71.54**	70.68	96.79	96.26	96.51	-	-	-	-	-	-
Vis-Phone	-	-	70.79	96.26	96.44	96.26	-	-	-	-	-	-
LEBERT	69.88	71.05	70.46	96.26	96.44	96.35	86.23	82.03	84.08	86.29	84.02	85.14
NER-ELF	70.85	70.47	70.66	96.04	97.02	96.53	-	-	-	-	-	-
CNEREA	**72.68**	70.69	**71.67**	96.60	**97.46**	**97.03**	**87.69**	**91.20**	**89.41**	**89.27**	**90.84**	**90.05**

The best results are shown in bold.

## Data Availability

The data presented in this study are available upon request from the corresponding author. The data are not publicly available due to copyright.
